# Supplementary material for: A First Methodological Development and Validation of ReTap: An Open-Source UPDRS Finger Tapping Assessment Tool Based on Accelerometer-Data
Source: Sensors (Basel). 2023 May 31;23(11):5238. doi: 10.3390/s23115238 (PMC10256040; doi:10.3390/s23115238)
Supplement: Supplementary file 1 [file sensors-23-05238-s001.zip › sensors-2398488-supplementary.pdf]

Supplementary Material belonging to "Development and validation of ReTap: an open-source model for automated UPDRS finger tapping assessment based on accelerometer-data" by Habets & Spooner et al.

Table S1. Patient demographics and clinical information.

Demographics (Mean SD)		
	**Berlin**	**Düsseldorf**
N	18	20
Age (yrs)	60.5 8.7	62.6 8.5
Sex (% females)	38.8	15
Time since diagnosis (yrs)	9.8 5.2	14.7 5.9
Time since DBS implantation (months)	6.7 6.1	49.4 15.8
UPDRS-III Med OFF, Stim OFF	36.8 14.0	39.8 11.3
UPDRS-III Med OFF, Stim ON	25.4 7.5	24.4 10.1
UPDRS-III Med ON, Stim OFF	29.6 14.5	28.2 12.1
UPDRS-III Med ON, Stim ON	29 8.8	13.7 7.8

Table S2. Predictive performance of different classification methodologies.

**Model**	**ICC-3k**	**Rho (Pearson's)**	**Mean error**	**Kappa**
WITH n-taps -> THREES				
Cross validation dvelopment, all taps included	0.59	0.42	0.67	0.28
Cross validation dvelopment, up to first 15 taps included	0.56	0.39	0.73	0.22
Holdout validation, all taps included	0.60	0.43	0.60	0.31
Holdout validation, up to first 15 taps included	**0.62**	**0.46**	**0.56**	**0.36**

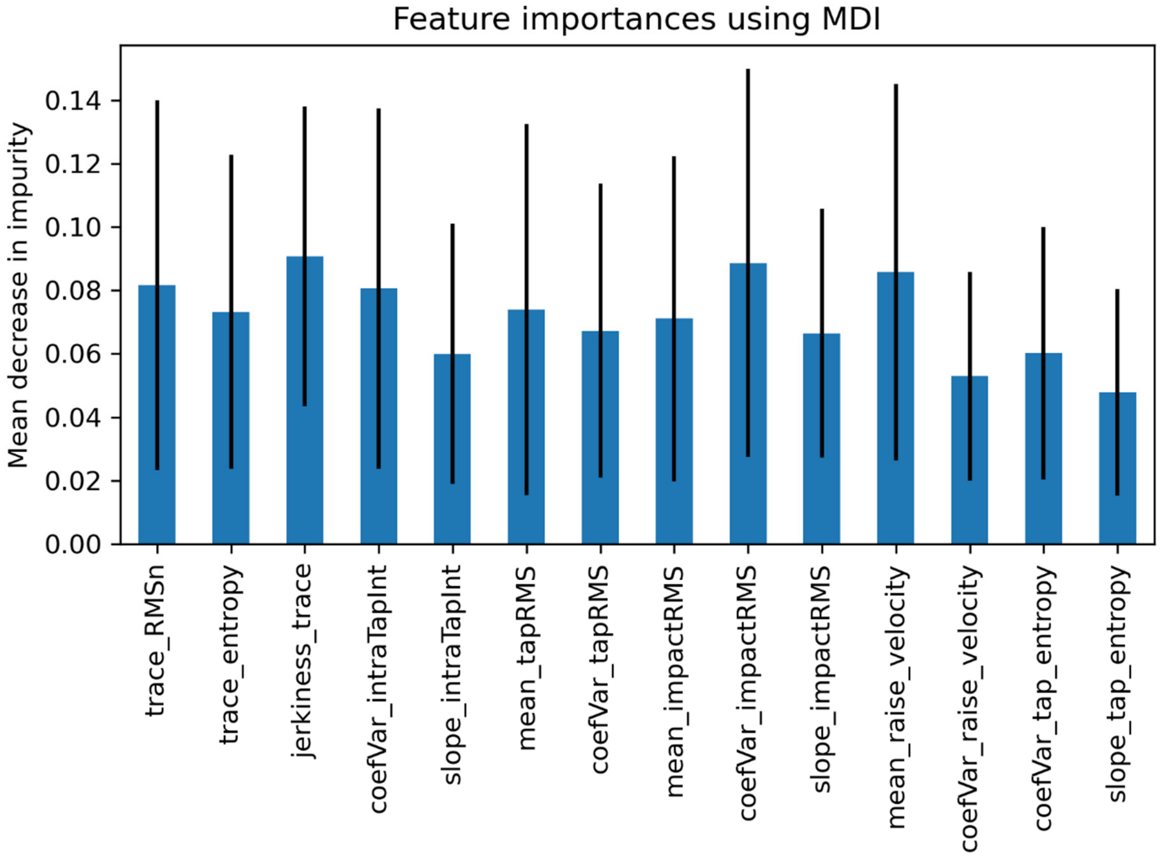

Figure S1: Classification feature importance. Feature importance in the final presented classification model, the random forest classifier considering up to the first 15 detected taps, calculated according to the Mean Decrease Impurity method.

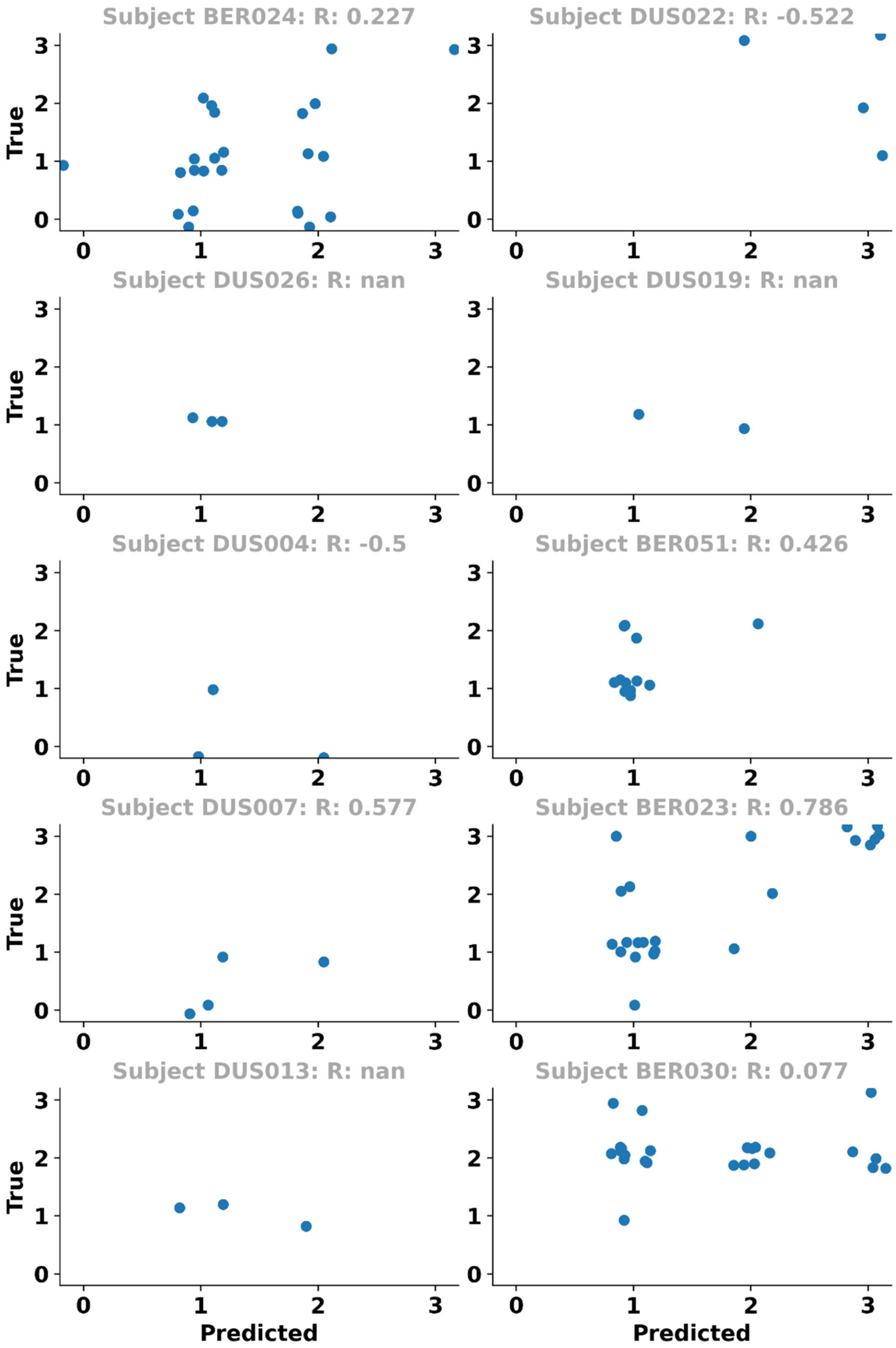

Figure S2: Individual predictive performance in holdout validation

. Scatterplots show correlation between predicted and true UPDRS tapping scores per subject included in the holdout validation.

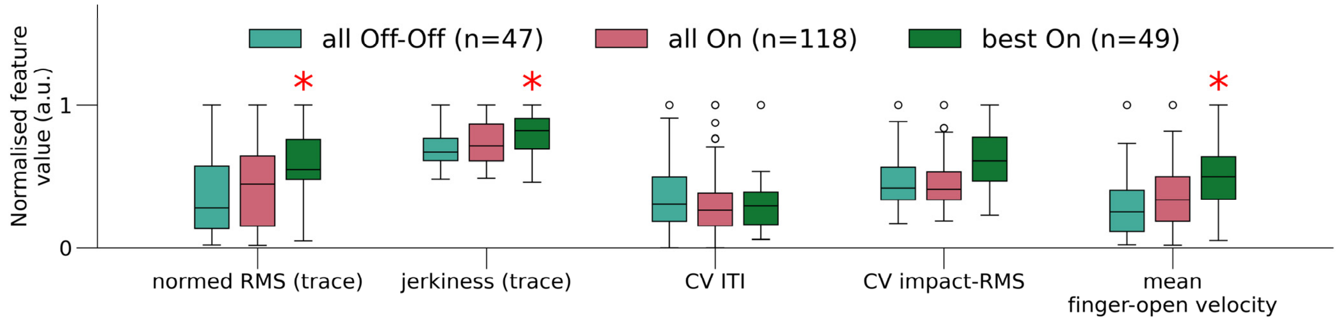

Figure S3: Group-level kinematic feature differences between therapeutic conditions. Feature values are shown for the five most important features (i.e., full block jerkiness, the coefficient of variation of impact-RMS, the mean raise velocity, the full block normalized RMS, and the coefficient of variation of ITIs) categorized per therapeutic condition. We included data performed with both hands (i.e., left and right finger tapping performance independently if both body sides were included from the subject) showing a mean improvement of more than 0.5 on the UPDRS Part III Item 3.4 task between medication-OFF, stimulation-OFF and one of the other therapeutic conditions (53%). Blue boxplots represent the medication-OFF, stimulation-OFF therapeutic condition, red boxplots represent all other conditions (medication-OFF, stimulation-ON and both medication-ON conditions), and green boxplots represent the individual *other* condition with the largest clinical improvement. The latter can be either medication-ON, stimulation-OFF; medication-ON, stimulation-ON; or medication-OFF, stimulation-ON. We chose the best clinical therapeutic condition per subject, as this can vary across individuals. The red asterisks indicate statistical significance compared to the medication-OFF, stimulation-OFF condition (MannWhitneyU analyses, p-value < 0.0002 (Bonferroni corrected, n=5)). Feature values were normalized only for visualization purposes. CV: coefficient of variation, ITI: inter-tap-interval, RMS: root mean square.

Normalized RMS values and mean finger-open velocities increased with better therapeutic conditions, which may be reflective of larger movement amplitudes when participants are under better therapeutic conditions. In contrast, while we expected the acc trace jerkiness to be higher during worse therapeutic conditions (i.e., medication OFF/stimulation OFF) potentially due to more hesitations present during the tapping block in PwP, the acc trace jerkiness was higher overall during the best therapeutic condition. This might be explained by a general increase in movement (i.e., greater tapping frequency) observed in PwP during better therapeutic conditions (e.g., stimulation ON sessions). Further refinement of this feature could improve the normalization of this jerkiness detection method, in order to account for the quantity of movements present during the tapping block. Similarly, the coefficient of variation (CV) for both ITIs and RMS values were expected to be higher when participants were under worse therapeutic conditions (e.g., medication OFF/stimulation OFF). However, this was only observed for the CV ITI. The higher movement amplitudes and tapping frequencies observed during better therapeutic conditions could possibly affect the CV RMS in this context.

Short overview of ReTap's repository workflow
https://github.com/jgvhabets/ReTap, v0.1-alpha

Quick overview of workflow

Finding your accelerometer data (DEFINE YOUR LOCAL FOLDER !!)

ReTap will find the (raw) accelerometer files you want to be processed in a FOLDER THAT YOU NEED TO DEFINE. This local folder-location you have to define within `ReTap/data/settings/configs.json`, as variable `raw_acc_folder`.

Executing ReTap to generate features and predictions

You can run ReTap's functionality either as a python-script directly from the command line, or execute it within a Jupyter Notebook. Both will be explained below.

Finding the results

ReTap will generate two folders containing the results and the illustrative figures of the feature extraction and the tapping-score-prediction. THESE FOLDER WILL BE CREATED NEXT TO THE FOLDER WITH RAW ACCELEROMETER DATA YOU DEFINED. These folders will be called `retap_results` and `retap_figures`.

```
.
├── YOUR DEFINED FOLDER (in configs.json)
├── retap_figures
│   └── block_detection
├── retap_results
│   └── extracted_tapblocks   (csv files with preprocessed data per detected tapping block)
│   └── features   (json-file with all features on a single-tap-event level, stored per detected tapping block)
│   └── predictions   (csv file with the predicted tapping-score, per detected tapping block)
```

Checking your results

There are some important steps you need to do, before you can work with the results.

- ReTap detects blocks of tapping events within the (raw) accelerometer data. All results are stored as 'blocks' resulting from a file. These blocks can be visually inspected in `retap_figures/block_detection`. Here you can identify if the block detection was successful, and you can decide which blocks you will include or discard.
